# Effect of supplementation of lecithin and carnitine on growth performance and nutrient digestibility in pigs fed high-fat diet

**DOI:** 10.14202/vetworld.2017.149-155

**Published:** 2017-02-07

**Authors:** Arathy Saseendran, K. Ally, P. Gangadevi, P. S. Banakar

**Affiliations:** Department of Animal Nutrition, College of Veterinary and Animal Sciences, Kerala Veterinary and Animal Sciences University, Mannuthy, Thrissur - 680 651, Kerala, India

**Keywords:** animal fat, carnitine, growth performance, lecithin, supplementation

## Abstract

**Aim:**

To study the effect of dietary supplementation of lecithin and carnitine on growth performance and nutrient digestibility in pigs fed high-fat diet.

**Materials and Methods:**

A total of 30 weaned female large white Yorkshire piglets of 2 months of age were selected and randomly divided into three groups allotted to three dietary treatments, T1 - Control ration as per the National Research Council nutrient requirement, T2 - Control ration plus 5% fat, and T3 - T2 plus 0.5% lecithin plus 150 mg/kg carnitine. The total dry matter (DM) intake, fortnightly body weight of each individual animal was recorded. Digestibility trial was conducted toward the end of the experiment to determine the digestibility coefficient of various nutrients.

**Results:**

There was a significant improvement (p<0.01) observed for pigs under supplementary groups T2 and T3 than that of control group (T1) with regards to growth parameters studied such as total DM intake, average final body weight and total weight gain whereas among supplementary groups, pigs reared on T3 group had better intake (p<0.01) when compared to T2 group. Statistical analysis of data revealed that no differences were observed (p>0.05) among the three treatments on average daily gain, feed conversion efficiency, and nutrient digestibility during the overall period.

**Conclusion:**

It was concluded that the dietary inclusion of animal fat at 5% level or animal fat along with lecithin (0.5%) and carnitine (150 mg/kg) improved the growth performance in pigs than non-supplemented group and from the economic point of view, dietary incorporation of animal fat at 5% would be beneficial for improving growth in pigs without dietary modifiers.

## Introduction

Swine industry has a major economic impact on agriculture throughout the world. Compared to other livestock species, pig rearing is considered to be more advantageous due to its low investment for farming, quick returns, better feed conversion efficiency, higher fecundity, short generation interval, and significance in improving socioeconomic status of weaker section of the society [1,2]. Pork is considered as the second most important meat in the world and is an excellent source of energy, protein, and vitamins. As per ICMR [3], out of 60 g of daily protein requirement, about 20 g should be from animal protein source. The global meat production is estimated to be 308.3 million tons including pork around 115 million tons [4]. Asia, the leading pork producing region account for 60% of total production. During 2012-13, our domestic production of pork was 0.45 million tons with an average meat yield of about 39 kg/animal, which is lower than the world average (79 kg/animal) [5,6].

Feed cost plays a major role in determining profitability of swine production and feed itself contributes 65-75% of total cost of production [6-8]. Cereal grains eventhough expensive comprise largest part of dietary energy in most swine diet. The major goal of nutritionist or swine producer should be to supply at feasible cost the nutrients at the right time in animal’s life [8,9]. Many alternative energy sources potentially cost-effective and useful in swine ration are produced by various industries. One such is animal fats which are a by-product of rendering process that includes lard, choice white grease, beef tallow, poultry fat which is said to contain 2.25 times metabolisable energy as compared to cereal grains [9,10]. Many researches indicate that addition of animal fat improves feed conversion efficiency, average daily gain, and increases feed palatability as well as reduces weaning stress to piglets [10,11].

In swine diet, fat utilization can be improved by feed additives such as lecithin and L-carnitine. For proper utilization of fat by animals, they should be digested and absorbed well in gastrointestinal tract [12,13]. Because fat is insoluble in water and difficult to handle in liquid medium emulsification is required for their digestion. Lecithin (phosphatidylcholine), a phospholipid extracted from soybeans are used as an exogenous emulsifier to enhance utilization of dietary fat [14]. Lecithin promotes incorporation of non-polar fatty acids of animal fat into the micellar phase and thereby increases the absorption of fat [14]. Carnitine is a quaternary amine compound. D- and L-carnitine are its two isomeric forms, of which most biologically active one is L-form [15]. Synthesis of carnitine take place mainly in liver and kidney and it depends on two precursors, methionine and L-lysine. For efficient transport of long-chain fatty acids into the compartment of mitochondria matrix from cytoplasm, carnitine is required and thus it improves utilization of fat [16]. This will lead to subsequent oxidation by the fatty acid oxidase complex for energy production; therefore, it plays a key role as energy regulator in tissues [16,17].

Addition of higher quantities of fat in the diet for pigs may cause oxidative stress and to prevent this stress antioxidant such as vitamin E (α-tocopheryl acetate) is to be included in the diet. Antioxidants will protect fat from oxidation and thereby prevent the production of rancidity substances [12,13]. Considering all these facts, this study was undertaken for determining the effect of dietary supplementation of lecithin and carnitine in weaned large white Yorkshire piglets fed high-fat diet on growth performance and nutrient utilization.

## Materials and Methods

### Ethical approval

The experimental design and plan of this study strictly followed the norms of the Institutional Animal Ethics Committee of Kerala Veterinary and Animal Sciences University (KVASU), Pookode, Kerala. Requisite permission for the selection of animals and laboratory analysis was granted by Academic Council and Institutional Animal Ethics Committee of KVASU, Pookode, Kerala.

### Study area

The study was conducted at Centre for Pig Production and Research, College of Veterinary and Animal Sciences, Mannuthy, Thrissur.

### Experimental design

A total of 32 weaned female large white Yorkshire piglets of 2 months of age were selected and randomly divided into three dietary groups as uniformly as possible with regard to weight. Each group had four replicates with two piglets per replicate in control group (T1) and three piglets per replicate in experimental groups (T2 and T3). All piglets were maintained under uniform management conditions throughout the experimental period of 98 days. Each piglet was fed with standard grower and finisher ration containing 18% and 16% of crude protein (CP) and 3265 kcal of metabolizable energy per kg of feed. The three groups of piglets were allotted to three dietary treatments as follows: T1 (control ration as per National Research Council [18]), T2 (control ration plus 5% animal fat) and T3 (T2 plus 0.5% lecithin and 150 mg/kg carnitine). All piglets were fed twice daily. Restricted feeding was followed by allowing them to consume as much as possible within the period of 1 h and balance feed, if any, were collected and weighed after each feeding.

### Data recording

Record of daily feed intake was maintained throughout the experimental period. Moisture content of both feed and leftover feed were analyzed to calculate total dry matter (DM) intake. The piglets were weighed at the beginning of the experiment and subsequently at fortnightly intervals to estimate total weight gain, average daily gain, and feed conversion efficiency, respectively. A digestibility trial was conducted toward the end of the experiment to determine the digestibility coefficient of the nutrients and availability of minerals like calcium and phosphorus by total collection method.

### Analytical procedures

Chemical compositions of feed and fecal sample were analyzed as per methods described in Association of Official Analytical Chemists [19].

### Statistical analysis

Data collected on various parameters were statistically analyzed by completely randomized design method as described by Snedecor and Cochran [20]. Means were compared by Duncan multiple range test using Statistical Package for Social Studies software. Ingredient and chemical composition of experimental grower and finisher rations are presented in Tables-1 and 2.

**Table-1:** Ingredient composition of experimental grower and finisher ration (%).

Ingredients	Experimental grower rations			Experimental finisher rations		
	
	T1	T2	T3	T1	T2	T3
Yellow maize	70	70	70	74	74	74
Wheat bran	1.5	1.5	1.5	3.6	3.6	3.6
Soyabean meal	26.25	26.25	26.25	20.5	20.5	20.5
Salt	0.5	0.5	0.5	0.5	0.5	0.5
Dicalcium phosphate	0.9	0.9	0.9	0.65	0.65	0.65
Calcite	0.85	0.85	0.85	0.75	0.75	0.75
Total	100	100	100	100	100	100
Animal fat (kg)	0	5	5	0	5	5
Lecithin^1^ (kg)	0	0	0.5	0	0	0.5
Carnitine^2^ (g)	0	0	15	0	0	15
Hyblend AB_2_D_3_K^3^ (g)	25	25	25	25	25	25
Becon-DS BE^4^ (g)	25	25	25	25	25	25
Zinc oxide^5^ (g)	45	45	45	30	30	30
Rovimix E-50^6^ (g)	10	10	10	10	10	10

1 Jubidol (Jubilant Life Science Ltd., Block 133, Village Samlaya Taluk-Sauli, Vadodara, Gujarat) contains optimal blend of lysophospholipids and phospholipids.

2 L-carnitine (Lot No-3-2014-001, Manufactured by Shanghai Kangxin Chemical Co-Ltd., China) containing lab graded L-carnitine.

3 Hyblend AB_2_D_3_K (Virbac Animal Health India Pvt. Ltd., Mumbai) contains: Vitamin A - 82,500 IU, vitamin B_2_ - 50 mg, vitamin D_3_ - 12,000 IU, Menaphthone sodium bisulphite - (vitamin K - Stabilized) - 10 mg.

4 Becon-DS BE (Varsha^®^ Multi Tech, KengeriHobli, Bengaluru) contains: Vitamin B1 – 8 mg, vitamin B6 – 16 mg, vitamin B12 – 80 mcg, vitamin E – 45 mg, calcium pantothenate – 30 mg, niacin – 120 mg, active live yeast – 600 MU, lactobacillus – 20 ms, folic acid – 2 mg, elemental calcium – 130 mg, elemental phosphorus – 6 mg.

5 Zinc oxide (Nice Chemicals Pvt. Ltd., Kochi) containing 81.38% of Zn.

6 Rovimix E-50 adsorbate (DSM Nutritional Products Ltd., Mumbai) contains 52.3% α-tocopheryl acetate (antioxidant)

**Table-2:** Chemical composition* of pig grower and finisher rations (%).

Parameters	Grower rations^1^			Finisher rations^1^		
	
	T1	T2	T3	T1	T2	T3
DM	90.01	90.26	90.35	89.05	89.92	89.92
CP	17.93	18.27	18.03	16.23	15.84	15.83
Ether extract	3.00	9.07	8.39	3.02	9.05	8.53
CF	2.48	2.29	3.31	2.51	2.54	3.19
Total ash	6.08	6.32	5.52	5.28	4.73	4.94
NFE	70.50	64.07	64.86	72.97	67.87	67.51
AIA	0.98	0.82	0.75	0.91	0.96	1.05
Calcium	0.74	0.62	0.63	0.75	0.65	0.64
Phosphorus	0.67	0.64	0.67	0.65	0.66	0.69
Gross energy (kcal/kg)	3938.06	4209.64	4111.40	3899.98	4163.24	4070.17

* On DM basis,

1 Mean of four values with SE. DM=Dry matter, SE=Standard error, AIA=Acid insoluble ash, CP=Crude protein, CF=Crude fiber, NFE=Nitrogen free extract

## Results and Discussion

Data on weekly average feed intake of pigs given the three experimental rations T1, T2 and T3 are presented in Table-3. The total feed intake recorded for the three treatments were 164.73, 177.34 and 181.04 kg, respectively. No significant difference was observed among treatment groups for average DM intake during 1^st^, 2^nd^ and 12^th^ week whereas supplementary groups (T2 and T3) showed similar and better intake than control group in all weekly intervals except 5^th^, 6^th^ and 9^th^ week where combination group (T3) showed higher intake than T1 and T2 group. This is in agreement with the findings of Overland and Sundstol [21] and Piao *et al*. [22] who had reported increased average DM intake in pigs fed diet incorporated with 5% and 6% tallow compared to control. Rincker *et al*. [17] observed a linear increase in feed intake by dietary incorporation of 50-100 ppm of L-carnitine along with 4-6% fat in pigs.

**Table-3:** Weekly average DM intake of pigs maintained on the three experimental rations (kg).

Week	Treatments^1^			p value

	T1	T2	T3	
1	9.02±0.12	9.04±0.07	9.06±0.06	0.96^ns^
2	9.14±0.05	9.82±0.16	9.96±0.16	0.31^ns^
3	9.29±0.37^a^	10.04±0.02^b^	10.37±0.16^b^	0.01*
4	9.95±0.10^a^	10.57±0.20^b^	10.93±0.17^bc^	0.004**
5	10.17±0.31^a^	11.22±0.13^b^	11.94±0.01^c^	0.00**
6	10.99±0.31^a^	12.28±0.08^b^	12.65±0.02^c^	0.00**
7	11.88±0.32^a^	13.06±0.30^b^	13.55±0.00^bc^	0.003**
8	12.63±0.20^a^	14.16±0.03^b^	14.15±0.05^b^	0.001**
9	13.01±0.01^a^	14.42±0.03^b^	14.52±0.01^c^	0.00**
10	13.28±0.19^a^	14.75±0.09^b^	14.87±0.02^b^	0.002**
11	13.67±0.10^a^	14.89±0.21^b^	15.05±0.05^b^	0.00**
12	13.58±0.12	14.32±0.72	14.81±0.20	0.24^ns^
13	14.10±0.14^a^	14.29±0.28^ab^	14.75±0.04^bc^	0.02*
14	14.02±0.15^a^	14.48±0.02^b^	14.43±0.02^b^	0.00**
Total feed intake	164.73±0.92^a^	177.34±1.58^b^	181.04±0.34^c^	0.00**

1 Mean of four observations with SE.

^a,b,c^Means having different superscripts within the same row differ significantly.

* Significant at 5% level (p<0.05),

** significant at 1% level (p<0.01), ns: Non significant, DM=Dry matter

L-carnitine serves as a co-substrate for the enzyme carnitine acyltransferase for reversible acetylation of coenzyme A and thereby acts as a carrier for transport of long-chain fatty acid from the cytosol into the inner mitochondrial membrane for undergoing β-oxidation of fatty acids [15,23]. Carnitine is adequately synthesized from precursor lysine, and hence, it is not considered as dietary essential for adult animals but for young ones attenuated de novo synthesis of carnitine [16] may necessitate dietary demand for carnitine [17]. Most animal products, including sow’s colostrum are a good source of carnitine, its greatest concentration is found in animal tissues, but they are poor in plant sources [15,24]. The primary biochemical mechanism of L-carnitine is that it forms esters with long chain activated fatty acids in the cytosol of the cells catalyzed by carnitine palmitoyl transferase Type Ι. These esters have the capacity to penetrate mitochondrial membrane [23,25]. Within mitochondrial membrane, the esters are cleaved off again from L-carnitine and fatty acids catalyzed by Type ΙΙ of carnitine palmitoyl transferase and this activated fatty acid released inside mitochondrion can be utilized for the production of energy. It is often classified as quasi-vitamin because of its essential role in fatty acid metabolism [26,27].

Danek *et al*. [28] suggested that dietary supplementation of lecithin at 0.1% level along with fat (1.3%) showed 4-9% improvement in total feed intake than the non-supplemented group. Several authors [7,21,29] reported that animal fat was found to be less digestible in young pigs compared to fats of vegetable origin. Cho *et al*. [30] revealed that utilization of rendered fat is limited due to its high long chain, saturated, non-polar fatty acids and the entry of these fatty acids into micellar phase is restricted, thereby reducing fat absorption. To improve absorption, lecithin (phosphatidylcholine) can be added as an emulsifier (phospholipids) which enhances the incorporation of non-polar fatty acids into micellar phase thereby increasing fat absorption in pigs [29]. It was observed that lecithin helps to improve diet’s fat digestibility by 3.1% and nutrient utilization [31]. For assisting transportation of lipids from the liver, lecithin is required thereby they can function as an energy source and it can also act as an emulsifier to support inadequate supply of bile acids secreted by the piglets [28,32].

The data with regard to fortnightly average body weight of pigs are presented in Table-4 and data regarding total weight gain, average daily gain and feed conversion efficiency of pigs during the overall period in Table-5. The statistical analysis of the data (Table-4) revealed that the supplementary groups (T2 and T3) were found to have significantly better average body weight when compared to control group (T1) in all fortnightly intervals. The present results are in agreement with Leibbrandt *et al*. [33] and Brumm and Peo [34] who had reported addition of tallow at 5% level improved final body weight of pigs linearly (p<0.01) compared with non-supplemented group. Reis *et al*. [29] observed 60% increase in body weight in pigs fed diet incorporated with 1.5% lecithin along with 6.5% tallow compared with control. Heo *et al*. [25] had reported a positive effect on body weight in pigs by adding 150 mg of L-carnitine per kg diet along with six per cent tallow. In contrary to the present findings, Mitchaothai *et al*. [11] reported that the average final body weight was not significantly different between pigs fed diet incorporated with beef tallow at 6% level. Chen *et al*. [35] reported no effect on body weight in finisher pigs by incorporating 250 mg of L-carnitine along with basal diet. Edward [36] reported that no significant effect was noted on final body weight by dietary incorporation of 0.5% lecithin in finisher pigs.

**Table-4:** Fortnightly average body weight of pigs maintained on the three experimental rations (kg).

Fortnight	Treatments^1^			p value

	T1	T2	T3	
0	21.59±0.06	21.78±0.12	21.76±0.08	0.74^ns^
1	27.39±0.12^a^	31.33±0.14^b^	32.04±0.27^bc^	0.00**
2	38.38±2.13^a^	49.00±2.30^b^	49.25±0.60^b^	0.004**
3	48.75±0.75^a^	57.46±2.29^b^	58.92±0.58^b^	0.002**
4	59.77±2.14^a^	68.79±2.03^b^	71.93±0.43^b^	0.001**
5	73.50±1.75^a^	81.84±2.92^b^	84.13±1.14^b^	0.04*

1 Mean of four observations with SE.

^a,b,c^Means having different superscripts within the same row differ significantly.

* Significant at 5% level (p<0.05),

** Significant at 1% level (p<0.01). ns=Non significant, SE=Standard error

**Table-5:** Total weight gain, average daily gain and feed conversion efficiency of pigs maintained on three experimental rations during the overall period.

Parameters	Treatments^1^			p value

	T1	T2	T3	
Average initial body weight (kg)	21.59±0.06	21.78±0.12	21.76±0.08	0.74^ns^
Average final body weight (kg)	73.50±1.75^a^	81.84±2.92^b^	84.13±1.14^b^	0.04*
Total weight gain (kg)	51.92±1.80^a^	60.06±2.82b	62.37±1.19^b^	0.04*
Average daily weight gain (g)	581.50±19.80	659.97±30.99	677.81±19.96	0.14^ns^
Total feed intake on DM basis (kg)	164.71±0.93^a^	177.31±1.58^b^	181.04±0.34^c^	0.00**
Feed conversion efficiency	3.18±0.10	2.97±0.12	2.91±0.06	0.35^ns^

1 Mean of four observations with SE.

^a,b,c^Means having different superscripts within the same row differ significantly.

* Significant at 5% level (p<0.05),

** Significant at 1% level (p<0.01). ns=Non significant, DM=Dry matter

Statistical analysis of data presented in Table-5 revealed that the treatment groups (T2 and T3) had significantly better total weight gain than the control group (T1) which are in accordance with the earlier reports of Baudon *et al*. [37] who had reported 5% increase in total weight gain by adding 6% tallow in the diet of pigs compared to control. Heo *et al*. [26] had observed improved total weight gain in pigs fed different level of protein along with 500 mg/kg L-carnitine in the diet of growing pigs compared to control. Todorova *et al*. [38] had reported a 6.5% increase in daily gain of pigs fed diet supplemented with 1% lecithin. The data also revealed that no differences were observed for average daily gain and feed conversion efficiency among treatment groups T1, T2 and T3. Min *et al*. [39] inferred that no significant difference on average daily gain and gain:feed ratio could be observed for those group of pigs fed on diet added with 5% fat. James *et al*. [23] observed no effect on average daily gain and gain:feed ratio by feeding L-carnitine at 50 mg/kg for pigs weighing 36-86 kg, respectively. No significant difference on average daily gain was reported by Papadopoulos *et al*. [40] by supplementing 0.5 g/kg lysolecithin along with 7 g/kg fat in the diet of weaned piglets.

**Figure-1:**
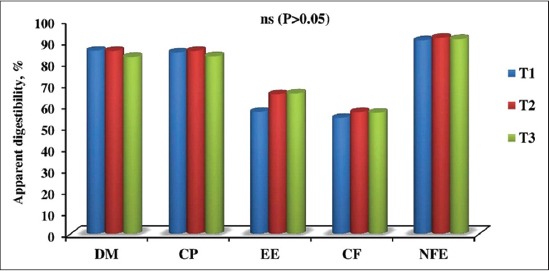
Apparent digestibility of nutrients of the three experimental rations, %. DM=Dry matter, CP=Crude protein, EE=Ether extract, CF=Crude fiber, NFE=Nitrogen free extract, ns=Non significant (p>0.05).

**Figure-2:**
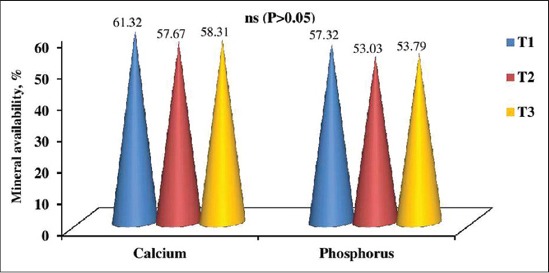
Availability of minerals of the three experimental rations (%). ns=Non significant (p>0.05).

The apparent digestibility of nutrients and availability of minerals in the experimental rations estimated from digestibility trial in pigs belonging to three dietary treatments are represented graphically in Figures-1 and 2. The digestibility of nutrients and mineral availability for three experimental rations were 82.75-85.76% for DM, 83.11-85.69% for CP, 57.17-65.76% for ether extract, 54.30-57.03% for crude fiber (CF), 90.52-91.82% for nitrogen-free extract, 57.67-61.32% for calcium, 53.03-57.32% for phosphorus, respectively. The digestible energy value for three dietary groups T1, T2 and T3 were 3509.57, 3729.11 and 3683.66 kcal/kg, respectively. No significant change (p>0.05) in the digestibility coefficient of nutrients and minerals was observed among the dietary groups which are in agreement with the earlier reports of Han and Thacker [16] who had observed no significant improvement in apparent digestibility of DM (81.2% versus 80.6%), CP (79.6% versus 76.8%), CF (42.4% versus 44.8%) when L-carnitine was added at the rate of 50 ppm in the diet of crossbred pigs compared to control. Kim *et al*. [14] reported that dietary supplementation of lecithin at 2.5% and 5% level along with 2.5% animal fat did not affect nutrient digestibility in finishing pigs. Huang *et al*. [41] noticed no differences on DM digestibility (81.90% and 81.92%) and nitrogen digestibility (80.88% and 79.54%) by adding 3.5% tallow along with the ration of crossbred pigs.

### Economics of gain

The cost of feed per kg for three rations (T1, T2 and T3) for the overall period was Rs. 24.38, 25.41 and 27.56, respectively. The cost of ingredients used for this study was as per the rate contract fixed for the supply of various feed ingredients to the farm for the year 2014-2015. The cost of feed per kg body weight gain of pigs maintained on the three dietary treatments was Rs. 77.37, 75.03 and 80.00 for the overall period and the values were statistically similar.

## Conclusion

The dietary incorporation of animal fat at 5% level or animal fat along with lecithin (0.5%) and carnitine (150 mg/kg) had significantly improved the total DM intake, average final body weight, and total weight gain in pigs than non-supplemented group but no differences were observed among them for average daily gain, feed conversion efficiency, and nutrient digestibility during the overall period. From the economic point of view, dietary supplementation of animal fat at five per cent level (T2) would be beneficial for improving the growth in weaned Large White Yorkshire pigs without dietary modifiers.

## Authors’ Contributions

KA was involved in the design of the study. AS carried out the experiment, collection, and analysis of the data and prepared the first draft of the manuscript under the guidance of KA. KA, PG revised the manuscript. PSB drafted and edited the manuscript.
